# Selective extraction and stabilization of bioactive compounds from rosemary leaves using a biphasic NADES

**DOI:** 10.3389/fchem.2022.954835

**Published:** 2022-08-12

**Authors:** Carolina Vieira, Sílvia Rebocho, Rita Craveiro, Alexandre Paiva, Ana Rita C. Duarte

**Affiliations:** LAQV@REQUIMTE, Departamento de Química, NOVA School of Science and Technology, Universidade NOVA de Lisboa, Caparica, Portugal

**Keywords:** natural deep eutectic systems, ultrasound-assisted extraction, *Rosmarinus officinalis*, antioxidants, rosmarinic acid

## Abstract

Rosemary (*Rosmarinus officinalis*) is a natural source of bioactive compounds that have high antioxidant activity. It has been in use as a medicinal herb since ancient times, and it currently is in widespread use due to its inherent pharmacological and therapeutic potential, in the pharmaceutical, food, and cosmetic industries. Natural deep eutectic systems (NADESs) have recently been considered as suitable extraction solvents for bioactive compounds, with high solvent power, low toxicity, biodegradability, and low environmental impact. The present work concerns the extraction of compounds such as rosmarinic acid, carnosol, carnosic acid, and caffeic acid, from rosemary using NADESs. This extraction was carried out using heat and stirring (HS) and ultrasound-assisted extraction (UAE). A NADES composed of menthol and lauric acid at a molar ratio of 2:1 (Me:Lau) extracted carnosic acid and carnosol preferentially, showing that this NADES exhibits selectivity for nonpolar compounds. On the other hand, a system of lactic acid and glucose (LA:Glu (5:1)) extracted preferentially rosmaniric acid, which is a more polar compound. Taking advantage of the different polarities of these NADESs, a simultaneous extraction was carried out, where the two NADESs form a biphasic system. The system LA:Glu (5:1)/Men:Lau (2:1) presented the most promising results, reaching 1.00 ± 0.12 mg of rosmarinic acid/g rosemary and 0.26 ± 0.04 mg caffeic acid/g rosemary in the more polar phase and 2.30 ± 0.18 mg of carnosol/g of rosemary and 17.54 ± 1.88 mg carnosic acid/g rosemary in the nonpolar phase. This work reveals that is possible to use two different systems at the same time and extract different compounds in a single-step process under the same conditions. NADESs are also reported to stabilize bioactive compounds, due to their interactions established with NADES components. To determine the stability of the extracts over time, the compounds of interest were quantified by HPLC at different time points. This allows the conclusion that bioactive compounds from rosemary were stable in NADESs for long periods of time; in particular, carnosic acid presented a decrease of only 25% in its antioxidant activity after 3 months, whereas the carnosic acid extracted and kept in the methanol was no longer detected after 15 days. The stabilizing ability of NADESs to extract phenolic/bioactive compounds shows a great promise for future industrial applications.

## 1 Introduction

Rosemary (*Rosmarinus officinalis*, Lamiaceae) is a green shrub plant that is originally from the Mediterranean region. It is a natural source of bioactive compounds, and its essential oil has powerful properties. It has been used since ancient times as a medicinal herb due to its healing properties for illnesses such as headache, dysmenorrhea, stomachache, epilepsy, rheumatic pain, spasms, nervous agitation, improvement of memory, hysteria, depression, and physical and mental fatigue (Borrás-Linares et al., 2014; Rahbardar and Hosseinzadeh, 2020). With its characteristic fragrance, rosemary can also be used as an aromatic plant in culinary or ornamental uses (Oliveira et al., 2019). Rosemary extract has been used for more than 20 years as a natural preservative in food due to his its antioxidant activity (Birtic et al., 2015). In 2010, the European Commission classified some of the antioxidant constituents of rosemary as food additives (E392), namely carnosic acid and carnosol (Commission Directives 2010/67/EU and 2010/69/EU). However, the antioxidant activity of rosemary has been noted not only in the food industry, but also in the pharmaceutical area, due to its inherent pharmacological and therapeutic potential. These compounds are currently present in several cosmetic formulas, as well as in pharmaceutical products (Neves et al., 2018; Oliveira et al., 2019; González-Minero et al., 2020).

Among the phytocompounds present in this plant, carnosic acid, rosmarinic acid, carnosol, and caffeic acid have been highlighted. Interactions between these compounds can promote several pharmacological effects (Oliveira et al., 2019). Carnosic acid can promote anti-inflammatory (Wang et al., 2018), antiproliferative (Bourhia et al., 2019), and antitumor activity (Allegra et al., 2020), and it has inhibitory effect on digestive enzymes (lipase, *a*-amylase, and *a*-glucosidase) (Ercan and El, 2018), a suppressive effect on lipogenesis (Song et al., 2018), and a protective effect on photoreceptor cells (Albalawi et al., 2018). Rosmarinic acid has neuroprotective (Cui et al., 2018), antiproliferative (Ma et al., 2018), and antiviral activity (Tsukamoto et al., 2018), and it can be used for anxiety control (Makhathini et al., 2018) and as complementary agent to anticancer chemotherapy (Radziejewska et al., 2018). Carnosol shows anti-inflammatory (Oliviero et al., 2018), antifungal (Ramírez et al., 2018), antiproliferative (Aliebrahimi et al., 2018), and antidiabetic (Samarghandian et al., 2017) activities, and it is also has protective against renal injury (Zheng et al., 2018). Caffeic acid has many health benefits, including antioxidant properties and anti-inflammatory, anticancer, and antiviral capacities (Huang et al., 2018; Monteiro Espíndola et al., 2019).

Because of these beneficial effects, the demand for bioactive rosemary compounds in the pharmaceutical, food, and cosmetic industries has increased, as has the value of the plant, making it in high demand. Over the last two decades, an average of 120 scientific papers have been published on rosemary per year has, clearly showing interest in this plant (Andrade et al., 2018). Several aromatic plants are cultivated all over the world (including rosemary), and increased production of it has also generated more residues. From a sustainability point of view, the unused residues should be further valorized. They are a source of bioactive compounds, and their recovery makes them a source of added-value products.

The extraction of bioactive compounds from rosemary is usually carried out with traditional volatile organic solvents derived from petroleum. These solvents are toxic to humans and are harmful to the environment, as well as causing high energy consumption. Furthermore, organic solvents can leave traces in the extract, which may alter its bioactive properties.

Deep eutectic systems (DESs) are promising alternative extraction solvents (Chemat et al., 2015). DESs can be defined as mixtures of two or more components, solid or liquid, which establish strong intermolecular interactions at a given molar ratio, essentially hydrogen-bond interactions, causing a melting point depression of the DES in regard to the individual components, leading to a liquid system (in some cases, liquid at room temperature) (Hansen et al., 2021). Moreover, when its constituents are primary metabolites, namely, amino acids, organic acids, alcohols, or sugars, they are termed as natural DESs (NADEs), which are in some cases biocompatible, more biodegradable, and with lower toxicity. As solvents, NADESs have advantages over conventional solvents, due to their adjustable viscosity, polarity, solubilization power, and negligible volatility (Paiva et al., 2014). The presence in NADESs of the functional groups of carboxyl and hydroxyl, as well as of amino acids , is responsible for the intermolecular interactions and some of its characteristics, particularly their solubilizing behavior and physicochemical properties.

NADESs demonstrate excellent results from extraction compared to conventional solvents (Liu et al., 2018). NADESs have been proven to have a high extraction capacity for phenolic compounds due to the interactions established between phenolics and NADES constituent groups. They also show a higher extraction yield than conventional solvents, such as water or ethanol (Dai et al., 2013a; Paiva et al., 2014). In addition, depending on the use and safety of the NADES applied, the extracts do not require post-extraction purification. Furthermore, keeping the extracts in the NADES can increase their shelf-life and bioactivity, promoting their stability (Dai et al., 2014). It is noteworthy that from an economic and environmental perspective, these systems present advantages concerning the simplicity of their preparation, their low cost, and their sustainability.

All of the studies of DESs as solvents in the extraction of polyphenols and flavonoids from rosemary are very recent. The most studied DESs are choline-chloride (ChCl)-based ones (Bajkacz and Adamek, 2018; Barbieri et al., 2020; Wojeicchowski et al., 2020, 2021; Calderón-Oliver and Ponce-Alquicira, 2021; Vladimir-Knežević et al., 2022). The use of ChCl combined with several compounds has been reported, for example, 1, 2-propanediol, reaching high levels of polyphenols (Barbieri et al., 2020; Wojeicchowski et al., 2021). Glyceline, or ChCl combined with glycerol (1:2), when used as a pretreatment in a proportion of 10% aqueous solution in the essential oil extraction from rosemary leaves, presents higher content of camphor, verbenone, and borneol and showed better antioxidant activity (Stanojević et al., 2021).

Extraction efficiency for the target compounds of rosemary has explored in more detail in hydrophilic DESs, particularly in ChCl-based DESs. The use of hydrophobic DES began later, with the aim of recovering fatty acids from aqueous media (Van Osch et al., 2015). Wang and his coworkers reported a study in which they compared DESs with different hydrophobicities, finding that hydrophobic DESs (e.g., menthol-based DESs) are more effective for the extraction hydrophobic compounds than hydrophilic DESs or traditional solvents (Wang et al., 2021). This indicates the infinite possibilities of DESs components and their combinations in promoting selectivity for the extraction and separation of certain bioactive compounds, as demonstrated with rosemary, as an example of a simultaneous extraction and fractionation of compounds with different polarities (Ali et al., 2019).

DES-based extraction has good extraction efficiency for bioactive compounds in plants (Ruesgas-Ramón et al., 2017; Tang and Row, 2020), although such extraction systems are ineffective for the simultaneous extraction of high-polarity and low-polarity compounds. Single-phase extractions with DESs are only able to extract compounds of similar polarity or analogues from plants (Cunha and Fernandes, 2018). In a work by Cao et al., a two-phase DES-based extraction is reported, in which a mixture of DESs of different polarities is used. This yielded a fractionated and selective extraction with a polar phase and a nonpolar phase, which extracted different compounds (Cao et al., 2018). Interestingly, it was also observed that two-phase systems could effectively enrich bioactive compounds with different polarities in the upper or lower phase, and the different phases could be easily separated after extraction process. These biphasic systems could act as a new paradigms in multicomponent extraction from plant residues, as well as from other different residues (Cao et al., 2018).

The purpose of this work is to extract compounds with added value from rosemary waste, using a more sustainable solvent such as a NADES. An initial screening established the optimal NADES extraction conditions, such as temperature, solid-liquid ratio, time, and extraction methodology. We explored the different types of NADESs with different compositions, and we identified the stability of the extracts provided by these systems. Furthermore, a fractionated extraction using a biphasic system composed by NADESs with different polarities was performed, promoting an easier and more efficient separation process in a single step. This strategy allows the selectivity of the target bioactive compounds for each NADES to be explored, which is extremely advantageous for specific applications.

## 2 Materials and methods

### 2.1 Plant material

The rosemary leaves were provided by Aromáticas vivas Ltd., from the residues of this company’s production. The plants were grown in Viana do Castelo (41°44′27.4″N 8°51′55.9″W), in the sub-region of Alto Minho in the northern region of Portugal, under optimal growth conditions defined by the company to meet high standards of food safety, quality, and sustainability. The initial water content of rosemary leaves was 63.07 ± 1.64%.

The water content in the rosemary leaves was monitored with a hygrometer during drying process using mass difference (KERN DAB 100-3, Germany). Drying was carried out at room temperature, until the mass stabilized. This process was carried out over a period of 2 months. In the end, the percentage of water was 8.79 ± 0.16%.

The plant material was ground (IKA tube-Mill control, Germany) to obtain fragments in the order of <0.5 mm in particle size (1 min at 1,200 rpm). The obtained rosemary powder was stored in plastic bags properly sealed and kept in a vacuum desiccator until further usage.

### 2.2 Chemicals

All chemical reagents were used as received after being purchased or kept in storage with no further treatment or purification. Lactic acid (≥85% purity), D-glucose monohydrate (≥97.5% purity), betaine (≥99% purity), DL-menthol (≥95% purity), myristic acid (≥98% purity), lauric acid (≥98% purity), D-sucrose (99.5% purity), D-sorbitol (98% purity), gallic acid (≥98% purity), and Nile red and rosmarinic acid were all purchased from Sigma-Aldrich (St. Louis, Missouri, United States). Citric acid monohydrate (99.5% purity) and Folin-Ciocalteau phenol reagent were obtained from Panreac (Barcelona, Spain). Glycerol (99.5% purity) and DL-malic acid (≥99% purity) were purchased from Scharlau (Barcelona, Spain). Ethylene glycol (≥99.5% purity) and ethanol (99% purity) were obtained from Carlo Erba (Val-de-Reuil, France). L-proline (99% purity) and β-alanin (99% purity) were obtained from Alfa Aesar (Haverhill, Massachusetts, United States). Sodium carbonate (99.5–100% purity) was purchased from Merck (Darmstadt, Germany), and methanol was acquired from Honeywell (New Jersey, United States). Carnosol, carnosic acid, and caffeic acid were acquired from Biosynth (Newbury, England). Trehalose (99% purity) was kindly provided by Hayashibara Co., Ltd. (Okayama, Japan).

### 2.3 Preparation of NADES

All NADESs were prepared using the heating/stirring method previously reported by Dai and co-workers (Dai et al., 2013b), taking into account a specific molar ratio (Table 1). The components in their respective molar ratios were heated and stirred until a clear liquid was formed. To avoid the degradation of the components, the temperature was maintained below 50°C, except for system proline and lactic acid (P:La (1:3)), prepared at room temperature.

**TABLE 1:** Composition, molar ratio, and physical appearance of the prepared NADESs.

NADES composition	Abbreviation	Molar ratio	Physical appearance
Betaine:ethylene glycol	B:EG	1:3	Transparent, colorless, slightly viscous liquid
Betaine:glycerol	B:Gly	1:2	Transparent, colorless viscous liquid
Citric acid:glycerol	Ca:Gly	1:1	Transparent, colorless viscous liquid
Citric acid:betaine	Ca:B	2:1	^a^
Lactic acid:glucose	La:Glu	5:1	Transparent, colorless liquid
Lactic acid:β alanin	La:Ba	1:1	Viscous white liquid
Lactic acid:betaine	La:B	2:1	Transparent, colorless liquid
Lactic acid:proline	P:La	3:1	Transparent, colorless liquid
Malic acid:sucrose	Ma:Su	1:1	^a^
Malic acid:glucose:glycerol	Ma:Glu:Gly	1:1:1	^a^
Malic acid:sorbitol	Ma:Sor	1:1	^a^
Menthol:lauric acid	Me:Lau	2:1	Transparent, colorless liquid
Menthol:myristic acid	Me:My	8:1	Transparent, colorless liquid
Menthol:lactic acid	Me:La	1:2	Transparent, colorless liquid
Trehalose:glycerol	T:Gly	1:30	Transparent, colorless slightly viscous liquid

a Systems that have not been successfully formed.

### 2.4 Characterization of NADES

#### 2.4.1 Determination of the water content

The water content of each NADES was determined by Karl-Fischer titration using an 831 KF Coulometer with generator electrode (Metrohm). The water content of the plant material was determined using a moisture analyzer DAB (Kern). The obtained values were provided as an average of three measurements.

#### 2.4.2 Determination of polarity by Nile red assay

The relative polarity of the prepared NADESs was obtained by determining the solvatochromic shift of the dye Nile red in ethanol, according to the procedure reported by Craveiro et al. (Craveiro et al., 2016), with slight modifications. The spectra were obtained using a UV-spectrophotometer (Thermo Scientific) measured at 400–800 nm at room temperature. Eq. 1 shows the polarity parameter calculated as molar transition energy (E_NR_) (Reichardt, 1994):

$$E_{\mathrm{NR}}=\frac{28591}{\text{λ}\max\left(nm\right)}$$ (1)

where E_NR_ is in kcal mol^−1^ and λ_max_ is the wavelength at the maximum absorbance in nm. All measurements were done in triplicate.

### 2.5 Extraction of bioactive compounds from rosemary leaves

NADES-based extraction was carried out the different conditions of heat and stirring extraction (HSE) and ultrasound-assisted extraction (UAE).

#### 2.5.1 Heat and stirring extraction

For HSE extraction, crushed dried rosemary leaves were mixed with the NADESs at different solid/liquid (S:L) weight ratios (1:20, 1:30, and 1:40). The extractions were performed in cycles of 15 min in a hotplate stirrer (Stuart heat-stir CB162, United States; AGIMATIC ED-C. J.P. Selecta, Spain) and stirred in a vortex for 1 min between cycles), according to procedures previously described by (Barbieri et al., 2020). There were two or four HSE cycles, lasting in 30 min and 1 h of extraction, respectively. The temperature was set to 40°C. The final extracts were centrifuged at 6,000 rpm for 20 min (Hermle), and the supernatant liquid was recovered and kept at 4°C. All of the extractions were done in triplicate.

#### 2.5.2 Ultrasound-assisted extraction

As with previous extraction methods, crushed dried rosemary leaves were mixed with NADESs at different S:L ratios (1:20, 1:30, and 1:40). The UAE extractions were performed in cycles of 15 min in an ultrasound bath (100 W) and frequency of 50–60 Hz (Grant XUB5, United Kingdom), and stirred in a vortex for 1 min between cycles, according to the procedure described previously (Barbieri et al., 2020). There were two or four UAE cycles in this part as well, resulting in 30 min and 1 h extraction, respectively. The selected extraction temperatures were 40°C and 60°C, chosen to evaluate the effect on extraction yield. The final extracts were centrifuged at 6,000 rpm for 20 min (Hermle) and the supernatant liquid was recovered and kept at 4°C. All extractions were done in triplicate.

#### 2.5.3 Soxhlet extraction

A mass of 2 g crushed dried rosemary leaves in a filter-paper bag was placed into the Soxhlet extraction chamber (250 ml) with 75 ml of methanol. The extraction was performed until the solvent was exhausted, and it was performed in triplicate. The residue was dried to remove the solvent at 40°C, and then it was weighed. The solvent in the solution was removed by evaporation, and the remaining solid (extract) was weighed and reserved.

#### 2.5.4 Biphasic extraction

Biphasic/fractionated extraction was performed using two NADESs of opposed polarities, yielding a biphasic system. The crushed and dried rosemary leaves were mixed with the biphasic system, and extractions were carried out with varied S:L ratios (1:20, 1:30, and 1:40) and temperatures (40°C and 60°C). All extractions were performed in triplicate.

### 2.6 Determination of total phenolic content by the Folin-Ciocalteau method

The total phenolic content was performed according to the colorimetric Folin-Ciocalteau method (Yılmaz et al., 2015), and it was calculated from a standard curve obtained with different concentrations of gallic acid (25–1,000 mg/L). In this method, 20 µl each of the diluted extracts was mixed with 1.58 ml distilled water and 100 µl Folin-Ciocalteu reagent. The mixture was vortexed and incubated at room temperature for 5–8 min. Following this, 300 µl of Na_2_CO_3_ saturated solution was added, and each sample was incubated at 40°C for 30 min. The absorbance of the samples was measured at 750 nm using a UV spectrophotometer (Thermo Scientific). All assays were performed in triplicate, and the concentration of the samples was determined and expressed in mg/L of gallic acid equivalents (GAE).

### 2.7 Quantification and characterization of extracted components

A high-performance liquid chromatography (HPLC) apparatus used was an Agilent 1,100 series separation module HPLC system (Agilent, Santa Clara, California, United States) equipped with a pump, an autosampler, a column oven, and a multi-wavelength detector. The analytical column was a Phenomenex Luna C18 (Phenomenex, 4.6 mm × 250 mm, 5.0 µm, Torrance, California, EUA). The column temperature was maintained at 30°C. All of the injected samples were previously diluted in a 1:10 ratio in ethanol. The mobile phase was composed of A (1% acetic acid in water) and B (methanol) with a gradient elution as follows: 0–20 min, linear from 10% to 65% B; 20–40 min, linear from 65% to 100% B; 40–45 min, maintained at 100% B; 45–47 min, linear from 100% to 10% B; and then finally, holding for 3 min. The mobile phase was filtered through a 0.22 µm membrane filter (Filter-Lab, Barcelona, Spain) and degassed in a vacuum. The flow rate was set at 1.0 ml/min, and the injection volume was 20 µl. The rosmarinic acid, carnosol, carnosic acid, and caffeic acid were determined at 284 nm. The data acquisition and remote control of the HPLC system were performed using OpenLAB CDS Chemstation edition software (Agilent, Santa Clara, California, United States).

### 2.8 Study of extract stability

The extracts obtained from the biphasic extraction that showed most promising results were used to determine the stability of the bioactive compounds in the NADESs. Extracts obtained from the Soxhlet were also studied for the sake of comparison. The extracts were stored protected from light and at room temperature. The extracts were left for 90 days under the conditions noted above. During this time, samples were collected taken at 1, 3, 7, 15, 30, 60, and 90 days, and the evaluation of the concentration of each compound was monitored, using HPLC.

### 2.9 Statistical analysis

All data was expressed as mean ± standard deviation of at least three independent experiments (n = 3). *p*-values lower than 0.05 (*p* < 0.05) were considered statistically significant (confidence interval of 95%). Statistical comparation of the means was made using the two-way ANOVA to investigate the statistical differences between the extractions and stability assays. Tukey’s test was used to perform the post hoc comparisons of the means.

All calculations were performed using the software GraphPad Prism 8.0.1 (San Diego, CA, United States).

## 3 Results and discussion

### 3.1 Characterization of NADES

Several factors can influence extraction efficiency using NADESs as solvents, such as their water content, inherent polarity and viscosity, and the hydrogen bond ratio (HBD:HBA).

To assess and evaluate potential systems for the extraction of phenolic compounds from rosemary, 10 NADESs were prepared and characterized. Table 2 presents the water content of these NADESs, as well as the viscosity values at 40°C.

**TABLE 2:** Water content and viscosity of the selected and previously prepared NADESs.

NADES	Water content (wt%)	Viscosity at 40°C (mPa.s)
Me:My (8:1)	0.03 ± 0.003	13.96 ± 1.10
Me:Lau (2:1)	0.09 ± 0.01	58.84 ± 1.70
B:Gly (1:2)	0.67 ± 0.05	606.36 ± 8.70
B:EG (1:3)	0.83 ± 0.01	35.69 ± 2.20
T:Gly (1:30)	1.42 ± 0.05	439.00 ± 2.30
Me:La (1:2)	7.34 ± 0.16	58.84 ± 1.70
La:B (2:1)	9.17 ± 0.13	374.60 ± 0.00
P:La (3:1)	10.25 ± 0.19	179.52 ± 0.10
Ca:Gly (1:1)	10.65 ± 0.85	1654 ± 26.50
La:Glu (5:1)	10.78 ± 0.13	42.94 ± 1.70

No water was added during the preparation of all of these NADESs. However, some NADES components contained water molecules in their composition, such as glucose and citric acid, which are both monohydrate, lactic acid, which has 15 wt% water content. Systems with menthol usually present a very low water content in their composition, being more hydrophobic and nonpolar.

**FIGURE 1:**
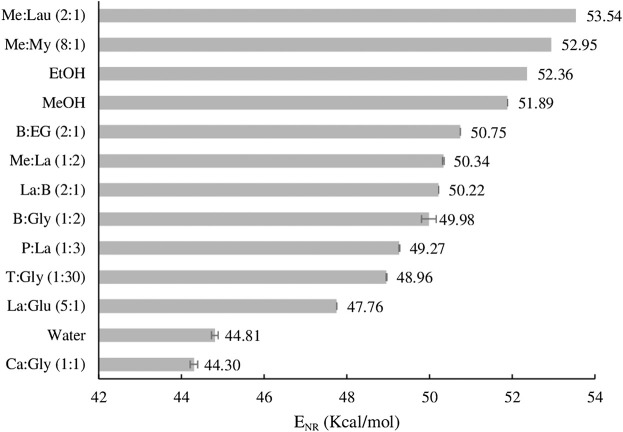
Relative polarity scale of the studied NADESs determined in ethanol, expressed as E_NR_ values.

Because the polarity of a solvent strongly impacts its solubilizing capacity (Dai et al., 2013b) and selectivity (Tang and Row, 2020), the polarity of the NADES was also measured (Figure 1).

As seen in Equation 1, lower E_NR_ values indicate higher polarity than the reference solvent (in this case ethanol). This implies that solvents with higher polarity change the dye’s λ_max_ to higher wavelength values, resulting in smaller E_NR_ values. From Figure 1, it is observed that the more polar NADESs is Ca:Gly (1:1), followed by La:Glu (5:1), and the less polar ones are Me:Lau (2:1) and Me:My (8:1). The results are in agreement with previous studies that have found that NADESs composed with organic acids tend to have a higher polarity (Dai et al., 2013b). In addition Dai et al. (2013b) reported that NADESs with amino acids and pure-sugar based, showed a polarity values closer to water (44.81 kcal mol^−1^), and finally, both sugar and polyalcohol based NADESs are less polar, with polarity values closer to the ones for methanol (51.89 kcal mol^−1^) and ethanol (52.36 kcal mol^−1^) (Dai et al., 2013b).

The polarities of NADESs are dependent on their composition and the character of its components, as well as on the amount of water present. Experimental studies previously reported by Craveiro et al. have explored the influence that the presence of water could have in shifting the polarity of the NADESs, showing that different water contents for the same NADESs result in small changes in the ENR value, such that the higher the water amount, the higher the polarity (Craveiro et al., 2016).

It was observed that viscosity is also dependent on the composition of the NADESs (Table 2). At 40°C, the NADESs that exhibit higher viscosity are those made with glycerol, being the highest value obtained for Ca:Gly (1:1). NADES viscosity decreases with increasing temperature and increasing water content (Liu et al., 2018), and the NADESs studied in this work show the same behavior. NADES viscosity influences the extraction efficiency, as it affects mass diffusion of the plant material and the NADES, so that NADESs presenting higher viscosities can yield lower extraction efficiencies.

### 3.2 Extract characterization

A Soxhlet extraction using a traditional organic solvent, methanol, was used to characterize the extract in terms of TPC and to quantify the four individual target compounds. The results obtained are presented in Table 3.

**TABLE 3:** Results of the TPC in mg GAE/g rosemary and for the rosmarinic acid, carnosol, carnosic acid, and caffeic acid in mg/g rosemary for the extract obtained with methanol. Data are expressed as mean ± SD.

TPC (mg GAE/g rosemary)	35.00 ± 7.35
Rosmarinic acid (mg/g rosemary)	1.36 ± 0.28
Carnosol (mg/rosemary)	7.28 ± 2.08
Carnosic acid (mg/g rosemary)	19.28 ± 3.00
Caffeic acid (mg/g rosemary)	0.28 ± 0.14

As can be observed (Table 3) the compounds rosmarinic acid, carnosol, carnosic acid, and caffeic acid amount to ∼80% of the total amount of phenolic compounds in rosemary.

### 3.3 Extraction of bioactive compounds from rosemary leaves

#### 3.3.1 NADES screening

Previously characterized NADESs were used to extract the same bioactive compounds to compare the results. The extraction conditions for NADESs were a S:L ratio of 1:20 at a temperature of 40°C for a period of 60 min. Furthermore, the extractions were performed using HSE and UAE. To evaluate the extraction efficiency of the four main compounds, rosmarinic acid, carnosol, carnosic acid, and caffeic acid, HPLC analysis was used to identify and quantify the target compounds. The HPLC results (Table 4) showed that the UAE method increases extraction efficiency of when compared with HSE. This is also in agreement with previously reported results that adopt this method for the extraction of phenolic compounds (Dai et al., 2013c; Radošević et al., 2016). The exception was system T:Gly (1:30), which revealed a higher quantity extracted of carnosol in HSE, the only compound detected in the extract, although it had a very low value compared to the other NADESs.

**TABLE 4:** Extraction amount of rosmarinic acid, carnosol, carnosic acid, and caffeic acid (mg/g rosemary), extracted with different NADESs under the same extraction conditions, S:L ratio of 1:20, 40°C, and 60 min, using two different techniques HSE and UAE. Results are expressed as mean ± SD. Statistically significant differences between the effect of the HSE and UAE using NADESs are represent by letters. Different letters indicate significant differences within each extraction technique.

Systems	HSE					UAE				
	Rosmarinic acid	Carnosol	Carnosic acid	Caffeic acid	Total	Rosmarinic acid	Carnosol	Carnosic acid	Caffeic acid	Total
La:Glu (5:1)	0.21 ± 0.07^a^	0.32 ± 0.06^a^	0.63 ± 0.07^a^	0.14 ± 0.05^a^	1.30 ± 0.22	0.28 ± 0.01^a^	0.46 ± 0.08^a^	1.36 ± 0.47^a^	0.16 ± 0.04^a^	2.26 ± 0.54
B:Gly (1:2)	n.d.	0.20 ± 0.09^a^	n.d.	n.d.	0.20 ± 0.09	n.d.	0.33 ± 0.07^a^	n.d.	n.d.	0.33 ± 0.07
T:Gly (1:30)	n.d.	0.31 ± 0.22^a^	n.d.	n.d.	0.31 ± 0.22	n.d.	0.10 ± 0.01^a^	n.d.	n.d.	0.10 ± 0.01
Me:My (8:1)	n.d.	0.59 ± 0.21^a^	1.03 ± 0.57^a^	n.d.	1.62 ± 1.31	n.d.	1.75 ± 0.27^ab^	6.61 ± 1.33^b^	n.d.	8.36 ± 3.44
Me:La (1:2)	0.77 ± 0.02^b^	n.d.	0.63 ± 0.05^a^	n.d.	1.40 ± 0.10	0.83 ± 0.06^ab^	4.87 ± 2.25^bc^	11.23 ± 0.74^c^	0.46 ± 0.01^a^	17.39 ± 5.00
Me:Lau (2:1)	n.d.	0.39 ± 0.03^a^	6.72 ± 0.45b	n.d.	7.11 ± 4.48	n.d.	1.57 ± 0.80^a^	8.26 ± 0.21^b^	n.d.	9.83 ± 4.73
La:P (3:1)	0.23 ± 0.01^a^	n.d.	n.d.	n.d.	0.23 ± 0.01	0.22 ± 0.02^a^	0.99 ± 0.22^a^	2.27 ± 0.37^a^	n.d.	3.48 ± 1.04
La:B (2:1)	0.16 ± 0.00^a^	n.d.	n.d.	n.d.	0.16 ± 0.00	0.17 ± 0.00^ad^	0.86 ± 0.10^ab^	2.07 ± 0.19^a^	n.d.	3.10 ± 0.96
B:EG (2:1)	0.19 ± 0.01^a^	0.75 ± 0.11^a^	n.d.	n.d.	0.94 ± 0.40	0.65 ± 0.44^ac^	2.45 ± 0.17^abc^	0.63 ± 0.38^a^	0.11 ± 0.06^a^	3.84 ± 1.02
Ca:Gly	n.d.	n.d.	n.d.	n.d.	n.d.	n.d.	n.d.	n.d.	n.d.	n.d.

n.d, not detected.

The NADES with the highest extraction efficiency was Me:La (1:2), as it extracted all compounds of interest with the highest yields. That is probably due to the composition of this NADES; menthol and lactic acid have different polarities, making the character of this system more amphiphilic. In fact, regarding the polarity results on Figure 1, Me:La (1:2) can be considered a NADES with low polarity. As noted, organic acid-based NADESs tend to have a higher polarity, and the alcohol-based ones are naturally less polar. In addition, this NADES has higher extraction yield than conventional extraction with Soxhlet.

NADES Ca:Gly (1:1) was discarded, as it exhibits low extraction yields. This system has a high viscosity, and as such, the mass transfer was compromised. Systems such as T:Gly (1:30) present lower viscosities but also did not show an extraction ability for the compounds of interest. All of these systems have glycerol in their composition, which can be responsible for their high viscosity (Table 2).

Taking into account all of the tested NADESs, the one that was able to extract all the target compounds was La:Glu (5:1) with either of the extraction techniques tested but rendering better results in UAE. Menthol-based NADESs could extract a higher concentration of the compounds, highlighting Me:La (1:2) with a strong input using UAE. Despite both, Me:Lau (2:1) and Me:My (8:1) reaching an excellent total extraction yield, these systems were not able to extract rosmarinic acid.

#### 3.3.2 Optimization of single-phase NADES extraction

The previous results made it possible to verify that the system that presented the higher extraction yield in terms of concentration of all the compounds was Me:La (1:2). However, the fact that the other menthol-based NADESs do not extract rosmarinic acid was very important for choosing the most promising NADES to optimized the process. Here, it is important to emphasize that our objective is not only the higher extraction yield but also to be able to combine it with the selectivity of the NADES regarding to our target compounds. From these results, we decided to choose two polar NADESs (La:Glu (5:1) and La:P (3:1)) and two nonpolar NADESs (Me:Lau (2:1) and Me:My (8:1)) for testing.

To study the influence of the S:L ratio in the extraction of these four compounds from rosemary, several extractions were performed, with S:L ratios of 1:20, 1:30, and 1:40. Extraction also varied, from 30 to 60 min of extraction, in mg of compound/g of rosemary.

Table 5 shows the concentration, in mg/g rosemary, of each compound of interest from rosemary, extracted using NADESs referred above, two polar systems (La:Glu (5:1) and P:La (1:3)), and two nonpolar systems (Me:Lau (2:1) and Me:My (8:1)), obtained from HPLC analysis.

**TABLE 5:** Effects of UAE extraction time (30 and 60 min) and S:L ratio (1:20, 1:30, and 1:40) on the extracted content of each compound of interest from rosemary: rosmarinic acid, carnosol, carnosic acid, and caffeic acid (in mg of compound/g of rosemary) at 40°C. The results are expressed as mean ± SD. Statistically significant differences between the ratios (1:20, 1:30, and 1:40) in each system are represented by letters. Different letters indicate significant differences within each system.

Systems/Time			40°C		
		Compounds	1:20	1:30	1:40
La:Glu (5:1)	30 min	Rosmarinic acid	0.20 ± 0.08^a^	0.17 ± 0.09^a^	0.04 ± 0.01^a^
		Carnosol	0.84 ± 0.01^a^	0.58 ± 0.13^a^	0.81 ± 0.03^a^
		Carnosic acid	3.81 ± 1.92^a^	2.99 ± 1.32^a^	2.69 ± 0.26^a^
		Caffeic acid	n.d.	n.d.	n.d.
	60 min	Rosmarinic acid	0.29 ± 0.08^a^	0.37 ± 0.06^b^	0.38 ± 0.21^b^
		Carnosol	0.65 ± 0.12^a^	0.78 ± 0.01^a^	1.05 ± 0.15^a^
		Carnosic acid	2.78 ± 0.70^a^	4.41 ± 0.73^a^	4.76 ± 0.403^a^
		Caffeic acid	0.16 ± 0.04^a^	0.08 ± 0.01^a^	n.d.
La:P (3:1)	30 min	Rosmarinic acid	0.13 ± 0.08^a^	0.07 ± 0.05^a^	0.28 ± 0.15^b^
		Carnosol	0.60 ± 0.02^a^	n.d.	0.79 ± 0.20^a^
		Carnosic acid	2.63 ± 0.39^a^	1.67 ± 0.21^a^	3.20 ± 0.18^a^
		Caffeic acid	n.d.	n.d.	n.d.
	60 min	Rosmarinic acid	0.23 ± 0.02^a^	0.24 ± 0.16^ab^	0.38 ± 0.04^b^
		Carnosol	0.99 ± 0.22^a^	0.80 ± 0.41^a^	1.51 ± 0.21^a^
		Carnosic acid	2.27 ± 0.38^a^	4.75 ± 2.39^a^	4.51 ± 0.91^a^
		Caffeic acid	n.d.	0.03 ± 0.01^b^	n.d.
Me:Lau (2:1)	30 min	Rosmarinic acid	n.d.	n.d.	n.d.
		Carnosol	1.99 ± 0.10^b^	1.42 ± 0.33^abc^	2.17 ± 0.44^ab^
		Carnosic acid	6.66 ± 1.90^ab^	5.65 ± 2.24^ab^	5.34 ± 1.52^a^
		Caffeic acid	n.d.	n.d.	n.d.
	60 min	Rosmarinic acid	n.d.	n.d.	n.d.
		Carnosol	1.57 ± 0.80^ab^	1.15 ± 0.26^ac^	1.83 ± 0.34^ab^
		Carnosic acid	6.72 ± 0.45^ab^	4.44 ± 1.11^a^	4.21 ± 0.91^a^
		Caffeic acid	n.d.	n.d.	n.d.
Me:My (8:1)	30 min	Rosmarinic acid	n.d.	n.d.	n.d.
		Carnosol	1.21 ± 0.28^ab^	1.09 ± 0.24^ac^	1.22 ± 0.16^a^
		Carnosic acid	6.26 ± 2.10^ab^	7.52 ± 1.40^ab^	6.57 ± 0.91^ab^
		Caffeic acid	n.d.	n.d.	n.d.
	60 min	Rosmarinic acid	n.d.	n.d.	n.d.
		Carnosol	1.75 ± 0.27^b^	1.05 ± 0.29^ac^	0.92 ± 0.34^a^
		Carnosic acid	6.61 ± 1.33^ab^	4.87 ± 2.04^a^	4.91 ± 1.67^a^
		Caffeic acid	n.d.	n.d.	n.d.

n.d, not detected.

Only La:Glu (5:1) and P:La (1:3) of the chosen NADESs were able to extract rosmarinic acid, which is in agreement with the previous data, related to their polarity. In Table 5, it is observed that in the extractions performed for 30 min, the yield of rosmarinic acid is lower. As Bajkacz et al. showed, longer extraction times tended to favor the extraction of polyphenols. Therefore, it is to be expected that at higher temperatures, NADES viscosity will decrease, which promotes mass transfer, facilitating the migration of the species into the solvent. This yield is also dependent on the S:L ratio from 1:20 to 1:40, with a higher rosmarinic acid extraction yield for 1:40. Because the enhanced solubilization capacity in the extraction of the compounds of interest is influenced by the level of solvent penetration into the system matrix, the penetration tends to be more effective when the density of the solvent is lower. It is interesting to note that rosmarinic acid is not extracted by the menthol-based NADES due to its polarity.

The compound carnosol is extracted by all tested NADESs, and the higher extraction yields are obtained when using Me:Lau (2:1) and Me:My (8:1). This may be due to favorable interactions established between the NADES and carnosol and to more compatible polarities. Considering extraction time and S:L ratio, extraction yields are higher for 30 min and for a S:L of 1:20.

Carnosic acid extraction shows the same tendency as carnosol, as well as being extracted by all the NADESs under study, but the higher extraction yields are also obtained by menthol based-NADESs that have lower polarities. Due to their molecular similarity, carnosic acid and carnosol have close relative solubilities. According to Wojeicchowski and his coworkers the relative solubilities of these biomolecules were lower after water addition, possibly due to their high level of hydrophobicity, which confirms these results (Wojeicchowski et al., 2021).

Caffeic acid is only extracted when with the use of lactic acid-based NADESs and for S:L values of 1:20 and 1:30.

Table 5 shows that it is possible for the tested NADES to present selectivity towards specific compounds, a very important feature when designing an extraction process.

#### 3.3.3 Biphasic NADES extraction

**FIGURE 2:**
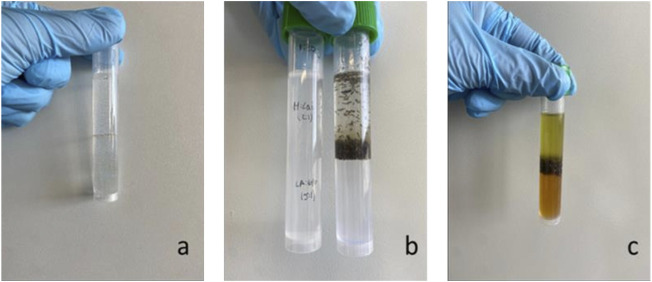
Biphasic system with nonpolar and polar NADESs **(A)**; biphasic system with nonpolar and polar NADESs before extraction **(B)** and after extraction **(C)**. System represented: Me:Lau (2:1) on top and La:Glu (5: I) below.

The conclusions of this work demonstrate that NADESs exhibit extraction selectivity. Hence, a different extraction strategy was tested and explored. By designing a NADES biphasic extraction system, it is possible to promote extraction efficiency and the further separation of compounds with different polarities (Tang and Row, 2020). Identifying the best choice of NADESs for each phase is a goal of this approach, where the miscibility between the two NADES phases should not occur, as seen in Figure 2A. This is made possible by combining NADESs with different polarities.

For this strategy, two polar systems (La:Glu (5:1) and P:La (1:3)) and two nonpolar systems (Me:Lau (2:1) and Me:My (8:1)) were chosen for the optimization of the biphasic UAE experiments. The extraction efficiencies of rosmarinic acid, carnosol, carnosic acid, and caffeic acid were tested under different S:L ratios at two temperatures (40°C and 60°C) over 60 min. The biphasic systems under study were combinations of the four NADESs, resulting in four combinations: 1) La:Glu (5:1)/Me:Lau (2:1), 2) La:Glu (5:1)/Me:My (8:1), 3) P:La (1:3)/Me:Lau (2:1) and 4) P:La (1:3)/Me:My (8:1).

Equal volumes of each NADES were used, and extractions were carried out. The two immiscible fractions were separated and analyzed individually. As can be observed in Figure 2C, a different color is observed in each phase of the extracts. This visual evidence shows that the different phases of the biphasic system are enriched in different compounds.

At 40°C, the most promising combination for extraction efficiency is system 1, with an S:L ratio of 1:30. This combination extracted 30% more of rosmarinic than the ratio 1:40 under the same conditions in the hydrophilic phase (La:Glu (5:1)).

Carnosic acid extracted in highest amount, with the highest values obtained in the nonpolar phase of the La:Glu systems (1 and 2), 12.34 ± 2.63 and 12.33 ± 1.18 mg/g rosemary, respectively. This compound exhibited similar behavior to carnosol concerning the S:L ratio, indicating higher concentrations at 1:30 and 1:40.

Carnosol was extracted at higher amounts in the following order of S:L ratio 1:40 > 1:30 > 1:20, analyzing the nonpolar fraction at 40°C. Rosmarinic acid was not detected in any nonpolar phase of the four systems, independent of temperature or ratio. This confirms the extraction selectivity of biphasic NADESs.

Systems 3 and 4 showed the highest extraction yields of caffeic acid in the polar phase, when extracted with an S:L ratio of 1:20 (∼0.12 mg/g rosemary), being the La:P (3:1), the most selective NADES towards this compound. When the P:La (1:3) was present in the combination of systems, caffeic acid showed a preferential tendency over the other solvents. In Table 6 the influence that the S:L ratio has on the extractability of this compound can be seen, such that it is higher for higher S:L ratios. Systems 1 and 2 revealed a low selectivity for caffeic acid, as only trace amounts were detected in both phases. In systems 3 and 4, caffeic acid was not detected in the nonpolar phase.

**TABLE 6:** Concentrations of rosmarinic acid, carnosol, carnosic acid, and caffeic acid (mg of compound/g rosemary) in the biphasic systems, varying the UAE S:L ratio (1:20, 1:30, and 1:40) and temperature (40°C and 60°C). Results are expressed as mean ± SD. Statistically significant differences between the ratios (1:20, 1:30, and 1:40) in each system for each compound are represented by letters. Different letters indicate significant differences within each system.

Systems			40°C			60°C		
		Compounds	1:20	1:30	1:40	1:20	1:30	1:40
1	La:Glu (5:1)	Rosmarinic acid	0.21 ± 0.13^a^	0.26 ± 0.10^a^	0.08 ± 0.02^a^	1.00 ± 0.12^a^	0.58 ± 0.09^a^	0.39 ± 0.06^a^
		Carnosol	0.27 ± 0.12^a^	n.d.	n.d.	0.32 ± 0.02^a^	0.31 ± 0.06^a^	0.26 ± 0.13^a^
		Carnosic acid	0.33 ± 0.10^a^	n.d.	0.63 ± 0.22^a^	0.83 ± 0.11^a^	0.52 ± 0.05^a^	0.70 ± 0.15^a^
		Caffeic acid	0.05 ± 0.00^a^	0.06 ± 0.02^a^	0.01 ± 0.00^a^	0.26 ± 0.04^a^	0.12 ± 0.02^a^	0.07 ± 0.02^a^
	Men:Lau (2:1)	Rosmarinic acid	n.d.	n.d.	n.d.	n.d.	n.d.	n.d.
		Carnosol	1.19 ± 0.34^b^	1.70 ± 0.33^a^	1.42 ± 0.37^a^	2.30 ± 0.18^b^	2.15 ± 0.04^b^	2.24 ± 0.10^b^
		Carnosic acid	7.71 ± 2.13^b^	12.34 ± 2.63^b^	9.60 ± 2.43^b^	17.54 ± 1.88^b^	14.49 ± 0.58^b^	13.23 ± 0.42^b^
		Caffeic acid	0.08 ± 0.00^b^	0.01 ± 0.00^b^	n.d.	0.09 ± 0.00^b^	0.09 ± 0.00^a^	n.d.
2	La:Glu (5:1)	Rosmarinic acid	0.18 ± 0.07^a^	0.25 ± 0.03^a^	0.17 ± 0.08^a^	0.82 ± 0.12^a^	0.51 ± 0.18^a^	0.50 ± 0.14^b^
		Carnosol	0.17 ± 0.04^a^	n.d.	n.d.	0.34 ± 0.04^a^	0.31 ± 0.04^a^	0.35 ± 0.01^a^
		Carnosic acid	0.40 ± 0.17^a^	0.28 ± 0.00^a^	0.99 ± 0.06^a^	0.90 ± 0.10^a^	0.91 ± 0.44^a^	0.48 ± 0.18^a^
		Caffeic acid	0.05 ± 0.00^ab^	0.05 ± 0.00^a^	0.01 ± 0.00^a^	0.19 ± 0.04^c^	0.11 ± 0.04^a^	0.08 ± 0.01^a^
	Me:My (8:1)	Rosmarinic acid	n.d.	n.d.	n.d.	n.d.	n.d.	n.d.
		Carnosol	1.04 ± 0.05^b^	1.65 ± 0.12^a^	1.73 ± 0.27^a^	2.23 ± 0.02^b^	2.03 ± 0.11^b^	2.36 ± 0.08^b^
		Carnosic acid	5.99 ± 0.59^b^	12.33 ± 1.18^b^	11.88 ± 2.24^b^	16.68 ± 0.48^b^	14.48 ± 0.33^b^	12.47 ± 0.57^b^
		Caffeic acid	0.07 ± 0.02^ab^	0.06 ± 0.01^a^	0.03 ± 0.01^a^	0.20 ± 0.02^ac^	0.09 ± 0.00^a^	0.12 ± 0.0^a^
3	La:P (3:1)	Rosmarinic acid	0.19 ± 0.12^a^	0.08 ± 0.07^b^	0.15 ± 0.09^a^	0.41 ± 0.05^b^	0.51 ± 0.16^a^	0.17 ± 0.09^c^
		Carnosol	0.60 ± 0.03^ab^	0.53 ± 0.17^b^	0.66 ± 0.36^b^	1.26 ± 0.07^bc^	1.05 ± 0.20^bc^	1.29 ± 0.20^bc^
		Carnosic acid	0.81 ± 0.00^a^	0.95 ± 0.42^a^	0.90 ± 0.11^a^	2.34 ± 0.05^a^	2.24 ± 0.29^abc^	1.30 ± 0.39^a^
		Caffeic acid	0.12 ± 0.02^bc^	0.06 ± 0.02^a^	0.03 ± 0.00^ab^	0.33 ± 0.01^cd^	0.20 ± 0.01^b^	0.12 ± 0.06^a^
	Men:Lau (2:1)	Rosmarinic acid	n.d.	n.d.	n.d.	n.d.	n.d.	n.d.
		Carnosol	0.95 ± 0.15^b^	0.95 ± 0.30^b^	1.13 ± 0.23^abc^	2.32 ± 0.03^b^	2.38 ± 0.25^bd^	2.02 ± 0.05^b^
		Carnosic acid	5.29 ± 1.16^b^	6.02 ± 2.43^bc^	7.21 ± 1.54^bc^	15.18 ± 0.31^bc^	15.84 ± 0.94^b^	11.34 ± 0.21^bc^
		Caffeic acid	n.d.	n.d.	n.d.	n.d.	n.d.	n.d.
4	La:P (3:1)	Rosmarinic acid	0.17 ± 0.07^a^	0.10 ± 0.04^b^	0.05 ± 0.02^a^	0.35 ± 0.03^bc^	0.38 ± 0.04^ab^	0.13 ± 0.05^cd^
		Carnosol	0.50 ± 0.04^ab^	0.43 ± 0.02^bc^	0.82 ± 0.25^b^	1.09 ± 0.14^bc^	1.13 ± 0.16^bc^	1.21 ± 0.11^bc^
		Carnosic acid	0.56 ± 0.06^a^	0.92 ± 0.10^a^	0.76 ± 0.12^a^	1.99 ± 0.36^a^	2.30 ± 0.08^abc^	1.08 ± 0.14^ac^
		Caffeic acid	0.11 ± 0.01^bc^	0.06 ± 0.03^a^	0.06 ± 0.01^b^	0.18 ± 0.00^c^	0.24 ± 0.05^bc^	0.10 ± 0.01^a^
	Me:My (8:1)	Rosmarinic acid	n.d.	n.d.	n.d.	n.d.	n.d.	n.d.
		Carnosol	0.85 ± 0.08^b^	0.43 ± 0.02^b^	0.82 ± 0.25^abc^	2.16 ± 0.16^b^	2.59 ± 0.25^bce^	2.11 ± 0.21^b^
		Carnosic acid	4.48 ± 0.72^bc^	6.55 ± 0.24^bc^	6.79 ± 0.51^bc^	14.75 ± 1.30^bc^	16.99 ± 1.05^bc^	12.51 ± 0.51^b^
		Caffeic acid	n.d.	n.d.	n.d.	n.d.	n.d.	n.d.

n.d, not detected.

At 60°C, La:Glu based systems (1 and 2) had the best results for the extraction of rosmarinic acid, ratio of 1:20.

Regarding carnosol, the systems that presented the highest yield were Me:My (8:1), with an S:L ratio of 1:30, from the nonpolar phase of the biphasic system 4 (2.59 ± 0.25 mg/g rosemary). However, compared with the results of Me:Lau (2:1) with an S:L ratio of 1:30 (2.38 ± 0.25 mg/g rosemary) and Me:My (8:1), with an S:L ratio of 1:40 (2.36 ± 0.00 mg/g rosemary), static analysis revealed no significant differences between these systems, being shown to be suitable for the extraction of carnosol.

At 60°C, the increased concentration of carnosic acid is evident, due to the nonpolar phase of all systems, in particular at the ratio 1:20.

Caffeic acid also showed an increased concentration with increased temperature. In general, the best results were observed for the ratio 1:20. Caffeic acid was not detected in the nonpolar phase of systems 3 and 4, similar to what occurred at 40°C.

The extraction efficiencies significantly changed with e increased temperature, as can be seen in Table 6. An increase in the concentration of bioactive compounds was observed for all systems at increases in temperature from 40°C to 60°C. These results also suggest an S:L ratio of 1:20 tends is the most suitable ratio in UAE biphasic extractions at 60°C.

In addition, all combinations showed selectivity, indicating that the bioactive compounds with variable polarity from rosemary were extracted selectively. Biphasic extractions of NADESs can not only extract with higher amounts of phenolic compounds but can also select compounds of different characters, based on their polarity. Compounds such as rosmarinic acid and caffeic acid were only extracted by nonpolar NADESs, while less polar compounds as carnosol and carnosic acid, were extracted in higher amounts by nonpolar NADESs.

### 3.4 Selectivity and partition coefficient

Partition separation processes are mainly used in the separation and purification of natural products. Both are based on same principle, which consists in the distribution of a solute immiscible compounds c in two phases composed of a mixture of solvents. The selection of the ideal two-phase system is the key, which must ensure an adequate distribution of the solute between the two liquid phases. In this case, we investigate four combinations of two NADESs that are immiscible to study the selectivity of four compounds which present different polarities.

To study the selectivity of the compounds between the two phases, the partition coefficient (*K*) was determined for each compound in the different systems (1–4), according to Eq. 2, for the different S:L ratios and temperatures.

$$K=\frac{{\left[C\right]}_{\left(nonpolar\;phase\right)}}{{\left[C\right]}_{\left(polar\;phase\right)}}$$ (2)

The results are presented in Table 7.

**TABLE 7:** Partition coefficient (*K*) of rosmarinic, acid, carnosol, carnosic acid, and caffeic acid in each biphasic system under different ratio conditions (1:20, 1:30, and 1:40) and temperatures (40°C and 60°C).

Systems	Compounds	40°C			60°C		
		1:20	1:30	1:40	1:20	1:30	1:40
La:Glu (5:1)/Me:Lau (2:1)	Rosmarinic acid	0	0	0	0	0	0
	Carnosol	4.41	0	0	7.19	6.94	8.62
	Carnosic acid	23.36	0	2.25	21.13	27.87	18.90
	Caffeic acid	1.64	0.22	0.25	0.35	0.78	0
La:Glu (5:1)/Me:My (8:1)	Rosmarinic acid	0	0	0	0	0	0
	Carnosol	6.12	0	0	6.56	6.55	6.74
	Carnosic acid	14.98	44.04	12.00	18.53	15.91	25.98
	Caffeic acid	0.18	1.25	2.94	1.07	0.84	1.44
La:P (3:1)/Me:Lau (2:1)	Rosmarinic acid	0	0	0	0	0	0
	Carnosol	1.58	1.79	1.71	1.84	2.27	1.57
	Carnosic acid	6.53	6.34	8.01	6.49	7.07	8.72
	Caffeic acid	0	0	0	0	0	0
La:P (3:1)/Me:My (8:1)	Rosmarinic acid	0	0	0	0	0	0
	Carnosol	1.70	2.35	1.24	1.98	2.29	1.74
	Carnosic acid	8.00	7.12	8.93	7.41	7.39	11.58
	Caffeic acid	0	0	0	0	0	0

The higher the partition coefficient of a compound, the higher the concentration of that compound in the nonpolar NADES in regards to the polar. Carnosic acid is the compound that presents the highest partition coefficient, followed by carnosol. This was expected, according to the previous results of concentration obtained by HPLC and due to the hydrophobic character of both compounds. The most suitable system for efficient and recovery of carnosol and carnosic acid is the combination of La:Glu (5:1), with Me:Lau (2.1) or Me:My (8:1). Due to the polarity of rosmaniric acid, the partition coefficient is zero for all systems, revealing that all rosmarinic acid that was extracted using this approach remains in the hydrophilic phase. The same occurs with caffeic acid but only in the La:P (3:1)-based systems, although in La:Glu (5:1)-based systems, the partition coefficient of this compound present lower values, showing a more hydrophilic character These results clearly show that biphasic systems based on La:P (3:1) are suitable for the efficient and sustainable recovery of rosmarinic and caffeic acids and La:Glu (5:1)-based systems reveled the best results for carnosol and carnosic acid. This shows that the lower the polarity of the compound, the higher the partition coefficient. For temperature and S:L, the best results were achieved at higher temperatures (60°C) and lower S:L (1:30/1:40).

The use of NADES biphasic systems allows the design of an effective extraction strategy in which each phase is enriched in the bioactive compounds with different polarities. In a single step, it is possible to selectively extract the target compounds and to modify with the temperature and S:L ratio at the same time. This strategy allowed to demonstrate that it is possible to obtain a single phase with no traces of rosmarinic acid.

### 3.5 Stability tests

Recently, it has been hypothesized that NADESs can have a stabilizing role towards certain molecules such as phenolic compounds and increase their stability for longer periods of time without losing their antioxidant activity. To evaluate the effect of NADESs on the stability on the obtained rosemary extracts for long periods of time, HPLC analysis of each fraction of the biphasic system was carried out.

The biphasic systems used for this study, were system 1 and system 4, with an S:L ratio of 1:20 and 1:30, respectively, at 60°C and with extraction time of 60 min.

#### 3.5.1 Quantification of bioactive compounds—HPLC

**FIGURE 3:**
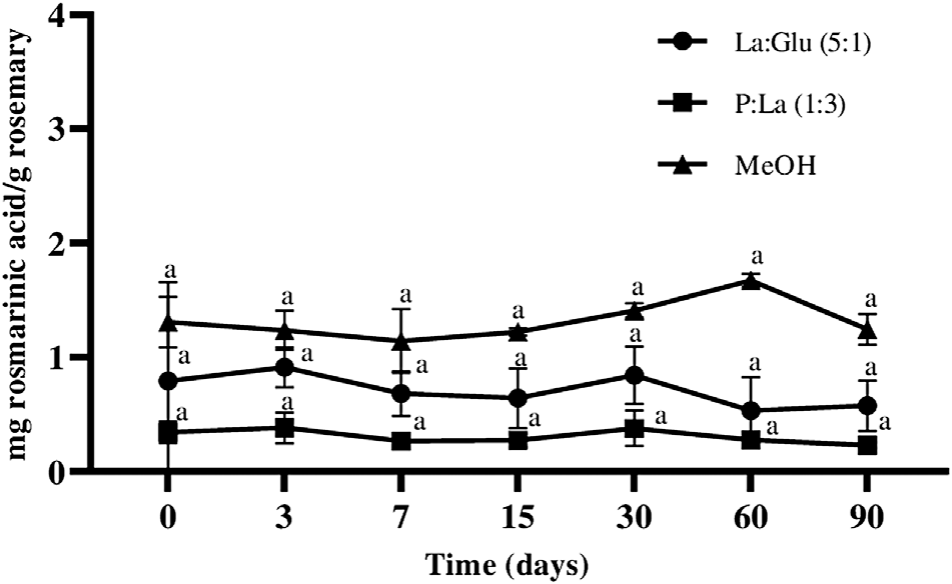
Influence of time on the content of rosmarinic acid extracted and kept in NADESs and Me0H. Values arc the average of three independent extracts replicates ±SD based on triplicate values. Statistically significant differences between the time points in each system are represented by letters. Different letters indicate significant differences within each system.

Over time, the concentration of rosmaniric acid in the extracts of La:Glu (5:1), La:P (1:3), and MeOH showed small variations (Figure 3). However, statistical analysis demonstrates no significant differences between the values of each time point were verified. These results show that rosmaniric acid is stable in NADES La:Glu (5:1) and P:La (1:3), at least for 90 days. Additionally, extracts obtained with MeOH, were also stable during the 90 days. Rosmarinic acid was not detected on nonpolar phases, Me:Lau (2:1) and Me:My (8:1), respectively.

**FIGURE 4:**
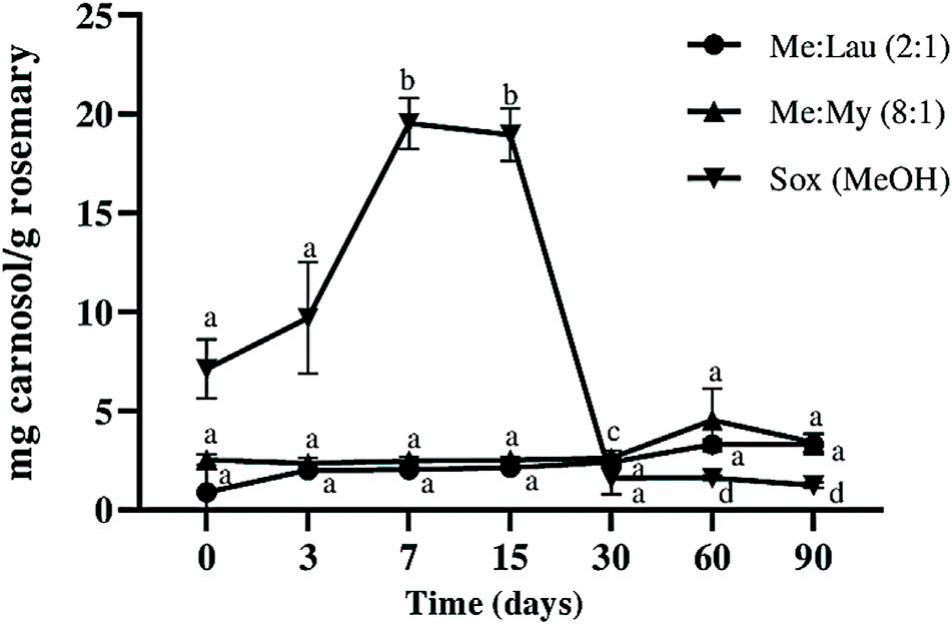
Influence of time on the extracted content of carnosol (C) in NADESs and methanol. Values are the average of three independent extracts replicates ±SD based on triplicate values. Statistically significant differences between the time points in each system are represented by letters. Different letters indicate significant differences within each system.

**FIGURE 5:**
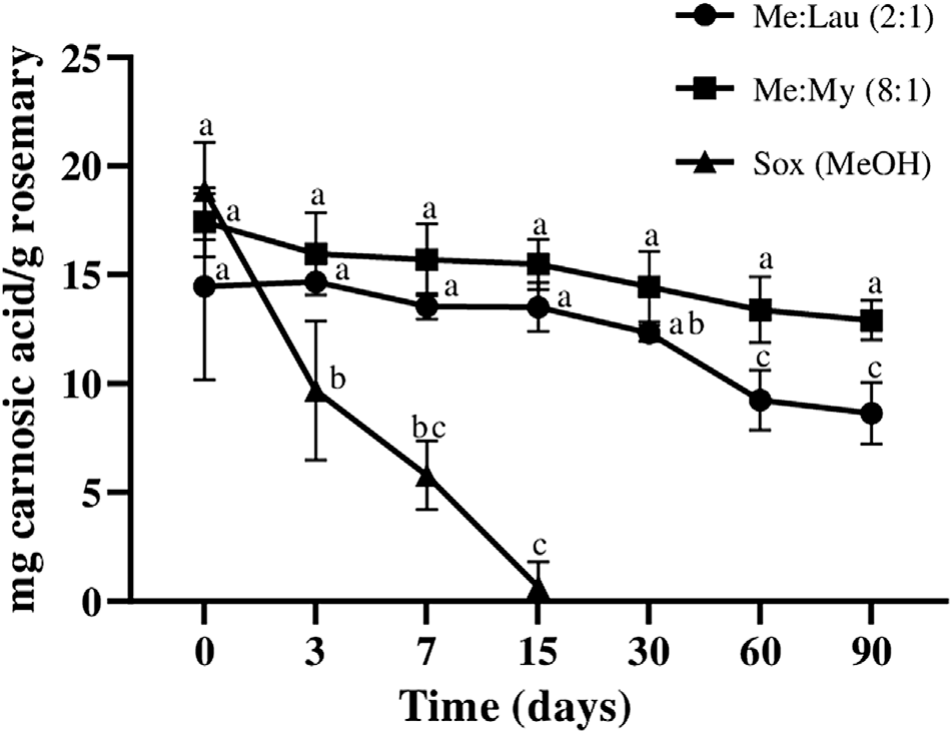
Influence of time on the extracted content of camosic acid (CA) in NADESs and methanol. Values are the average of three independent extracts replicates ±SD based on triplicate values. Statistically significant differences between the time points in each system are represented by letters. Different letters indicate significant differences within each system.

Carnosol is a derivative of carnosic acid when oxidized. This is a very common occurrence in plants under stress conditions. As a result, this may cause a strong decrease in carnosic acid and consequent accumulation of carnosol in the obtained extracts (Loussouarn et al., 2017). This can be correlated with the results obtained in this study. As we can observe in Figure 4 and Figure 5 there is a decrease in carnosic acid amount, and consequently an increase in carnosol. In the MeOH extracts this degradation was clearly more evident. At day 7, there was a loss of ∼72% of the carnosic acid, which caused an increase of ∼63% of carnosol (statistical difference *p* ≤ 0.001). Carnosol was not detected on La:Glu (5:1). The other NADESs showed a potential stabilizing ability for this compound, since its concentration remained constant during 90 days and no significant differences were verified by statistical analysis.

Carnosic acid content in the MeOH extract decreased over time, until it was not detected after 30 days (Figure 5), and after 15 days a decrease of almost 100% was already observed (statistical difference *p* ≤ 0.0001). The amount of carnosic acid remained stable in both Me:Lau (2:1) and Me:My (8:1) extracts after 15 days. In Me:My (8:1) remains stable until day 60 (no statistical differences were verify), on the other hand for Men:Lau (2:1), statistical analysis revealed a small difference after day 30 (*p* ≤ 0.05), maintained stable until day 90.

These NADESs showed to be appropriate for the stabilization of this bioactive compound. Carnosic acid was not detected on P:La (1:3) and in La:Glu (5:1) extracts, only a trace was detected, being nonsignificant when compared with the other systems.

**FIGURE 6:**
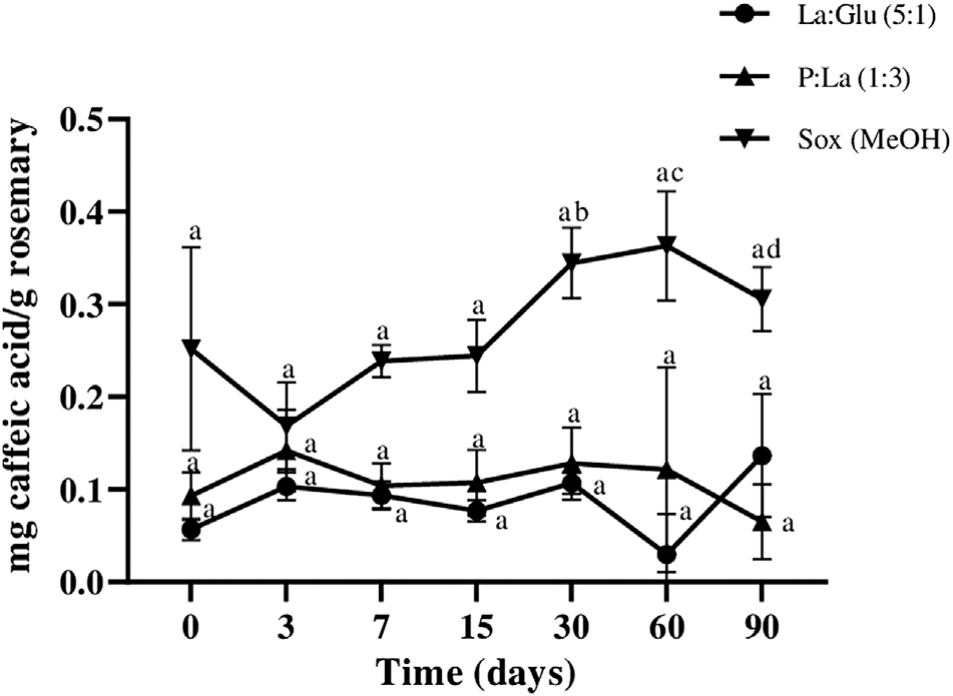
Influence of time on the extracted content of caffeic acid in NADESs and methanol. Values are the averages of three independent extract replicates ±SD based on triplicate values. Statistically significant differences between the time points in each system are represented by letters. Different letters indicate significant differences within each system.

Regarding caffeic acid content, Figure 6 shows that all extracts kept the compound stable for 90 days, except the one obtained with MeOH, which showed some fluctuations, as confirmed by statistical analysis. Thus suggests that the NADESs of P:La (1:3), La:Glu (5:1), and Me:Lau (2:1) provide good stability. However, the amounts of caffeic acid detected in these extracts were very low. Caffeic acid was not detected in Me:My (8:1).

Biphasic extracts of NADESs with different polarities had the ability to stabilize the bioactive compounds with the same character. In a general way, the four extracted bioactive compounds from rosemary under this study remained stable for at least 90 days with NADESs and presented a greater stability capacity than MeOH, in particular for carnosic acid.

The stabilization capacity of NADESs may have a direct association with viscosity. The viscosity of NADESs restricts the movement of molecules inside the extracts and allows more stable molecular interactions between the solvent and compounds, which prevents the degradation of the bioactive compounds extracted.

## 4 Conclusion

In this work, a methodology was proposed to use different NADESs as solvents to extract different compounds of interest from rosemary.

In the first approach, a screening of 10 NADESs at fixed extraction parameters was carried out. For comparison, conventional extractions using methanol were also performed. This study showed that NADESs composed of lactic acid (La:Glu (5:1) and P:La (1:3)) extracted higher amounts of bioactive phenolic compounds. In addition, Me:La (1:2) was the best NADES for rosmarinic acid, carnosol, carnosic acid, and caffeic acid. extraction (S:L ratio of 1:20 at 40°C performed for 60 min). UAE showed an increase in extraction yield when compared to the extractions performed by heat and stirring.

In addition, the optimization of extraction parameters revealed that an extraction time of 60 min was more efficient in the extraction of phenolic compounds.

The studied NADESs showed selectivity, according to their polar or nonpolar character and affinity to the compounds to be extracted. For the extract of Me:Lau (2:1), carnosic acid and carnosol had the predominant affinities. Rosmarinic acid was not detect for this extract, which indicated the selectivity of this NADES for nonpolar compounds.

Therefore, taking advantage of NADES extraction selectivity, a new approach was tested—an extraction with two-phase systems, where two immiscible DESs are used for extraction, and each phase will selectively extract the compounds of interest. From the results, it was possible to conclude that the biphasic NADES system can effectively increase the extraction yield and select compounds based on their polarity. As bioactive compounds with different polarities were found in the extraction phases with opposite polarities; for example, rosmarinic acid was not extracted by nonpolar systems but was detected in polar ones. The four studied bioactive compounds present in rosemary and were identified in extracts from biphasic system composed by La:Glu (5:1)/Me:Lau (2:1), with an S:L ratio of 1:20 at 60°C. This shows that biphasic NADESs are efficient for the extraction of phenolic compounds from rosemary. The results also show that NADESs present selectivity towards specific compounds and that using a biphasic system composed of NADESs of different polarities allows the simultaneous selective extraction of different compounds, facilitating its additional separation.

Following the stability of the four compounds of interest over time, a higher stability is observed in the NADESs La:Glu (5:1), Me:Lau (2:1), La:P (1:3), and Me:My (8:1) relative to MeOH. These NADES extracts remained stable up to 3 months, which shows that the use of NADESs as extraction solvents may not require the separation of the solvent from the extract, as NADESs improve extract stability.

This extraction strategy presents advantages over the conventionally used extraction methods and can play a crucial role in industrial applications and when scaling-up the process is envisaged. The extracts obtained with NADESs present the advantages of being used directly without additional purification processes and without the addition of preservatives. This provides the extracts several benefits for further applications in the pharmaceutical, cosmetic, or food industry, according to the therapeutic properties of the compounds present in these extracts.

## Data Availability

The raw data supporting the conclusions of this article will be made available by the authors, without undue reservation.
